# RapidArc, intensity modulated photon and proton techniques for recurrent prostate cancer in previously irradiated patients: a treatment planning comparison study

**DOI:** 10.1186/1748-717X-4-34

**Published:** 2009-09-09

**Authors:** Damien C Weber, Hui Wang, Luca Cozzi, Giovanna Dipasquale, Haleem G Khan, Osman Ratib, Michel Rouzaud, Hansjoerg Vees, Habib Zaidi, Raymond Miralbell

**Affiliations:** 1 Department of Radiation Oncology, University Hospital of Geneva, Geneva, Switzerland; 2 Oncology Institute of Southern Switzerland, Medical Physics Unit, Bellinzona, Switzerland; 3 Institute of Radiology Jean Violette, Geneva, Switzerland; 4 Department of Nuclear Medicine, University Hospital of Geneva, Geneva, Switzerland; 5 Faculty of medicine, UNIGE, University of Geneva, Switzerland

## Abstract

**Background:**

A study was performed comparing volumetric modulated arcs (RA) and intensity modulation (with photons, IMRT, or protons, IMPT) radiation therapy (RT) for patients with recurrent prostate cancer after RT.

**Methods:**

Plans for RA, IMRT and IMPT were optimized for 7 patients. Prescribed dose was 56 Gy in 14 fractions. The recurrent gross tumor volume (GTV) was defined on ^18^F-fluorocholine PET/CT scans. Plans aimed to cover at least 95% of the planning target volume with a dose > 50.4 Gy. A maximum dose (D_Max_) of 61.6 Gy was allowed to 5% of the GTV. For the urethra, D_Max _was constrained to 37 Gy. Rectal D_Median _was < 17 Gy. Results were analyzed using Dose-Volume Histogram and conformity index (CI_90_) parameters.

**Results:**

Tumor coverage (GTV and PTV) was improved with RA (V_95% _92.6 ± 7.9 and 83.7 ± 3.3%), when compared to IMRT (V_95% _88.6 ± 10.8 and 77.2 ± 2.2%). The corresponding values for IMPT were intermediate for the GTV (V_95% _88.9 ± 10.5%) and better for the PTV (V_95%_85.6 ± 5.0%). The percentages of rectal and urethral volumes receiving intermediate doses (35 Gy) were significantly decreased with RA (5.1 ± 3.0 and 38.0 ± 25.3%) and IMPT (3.9 ± 2.7 and 25.1 ± 21.1%), when compared to IMRT (9.8 ± 5.3 and 60.7 ± 41.7%). CI_90 _was 1.3 ± 0.1 for photons and 1.6 ± 0.2 for protons. Integral Dose was 1.1 ± 0.5 Gy*cm^3 ^*10^5 ^for IMPT and about a factor three higher for all photon's techniques.

**Conclusion:**

RA and IMPT showed improvements in conformal avoidance relative to fixed beam IMRT for 7 patients with recurrent prostate cancer. IMPT showed further sparing of organs at risk.

## Background

Biochemical failures (BF) of prostate cancer after external beam radiation therapy (RT) is not an unusual event and is observed in a substantial number of prostate cancer patients [1,2]. CapSURE™ (Cancer of the Prostate Strategic Urologic Research Endeavor) data have demonstrated a biochemical failure rate following radiation therapy as high as 63% [3]. Up to 70% of these patients will have evidence of recurrent or residual disease within the prostate gland [4]. Although curative treatment is still an option if the patient presents organ-confined disease only, no consensus exists however on the optimal salvage therapy modality for these patients. Therapeutic management of these patients includes salvage radical prostatectomy, cryotherapy, brachytherapy or high-intensity focused ultrasound, with or without hormonal deprivation therapy. Re-irradiation with conformal techniques is yet another strategy with potential curative intent. Re-irradiation techniques must however minimally deliver radiation dose to pre-irradiated organ at risk (OARs) in the direct vicinity of the target volume.

The demonstration of organ-confined only recurrent disease in patients with BF is not easily done with conventional radiology. Identifying precisely the target recurrent volume is of paramount importance when delivering focused high-radiation dose in a pre-irradiated area. Recent progress in imaging with PET tracers such as acetate or choline labelled with ^11^C or ^18^F have improved significantly the accuracy in diagnosing the site of relapse [5]. Local tracer uptake within the gland may correspond to the locally recurring gross-tumor volume (GTV) and can be contoured in the RT treatment planning system.

RapidArc (RA), is a novel technique which may achieve several objectives: i) improve organ at risks (OARs) and non-target tissue sparing compared to other intensity modulated RT (IMRT) techniques; ii) maintain or improve the same degree of target coverage; iii) reduce significantly the treatment time per fraction. Dose comparative studies using RA, have been published in prostate [6,7], cervix uteri [8] and anal canal cancer [9], showing significant improvements when compared to non-RA techniques. This technique could be thus used to treat geometrically complex partial recurrent tumor volumes within the prostate gland after RT.

The present study was undertaken to assess the treatment planning inter-comparison between photon and proton RT, namely IMRT and IMPT, to RA, as applied to a total of 7 recurrent pre-irradiated prostate cancer patients

## Methods

**Figure 1:**
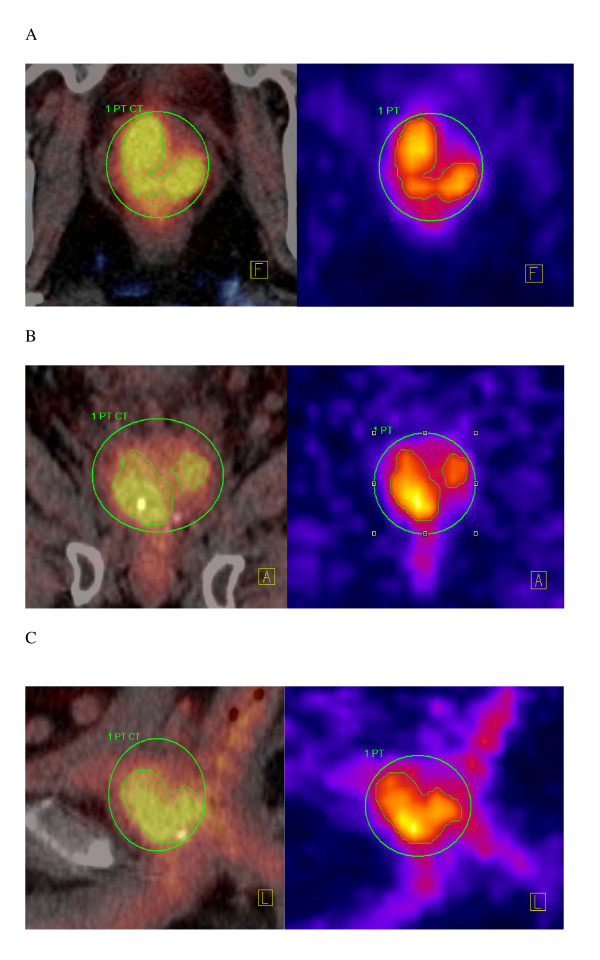
**GTV in the axial (A), coronal (B) and sagital (C) simulation CT with PET fusion and ^18^F-choline PET slice, respectively**.

The institutional ^18^F-Choline database containing 47 prostate cancer patients was queried to identify individuals with: 1) biochemically recurrence; 2) local relapse only; 3) previous high-dose (≥ 70 Gy) RT and 4) endorectal MRI. Seven of such patients were identified (median age, 77 years; Table 1). They all underwent previous curative 3D conformal RT (median dose, 74 Gy; HDR brachytherapy boost 14 Gy in 2 fractions, 2 patients), 4.8 to 7.6 (median, 5.9) years before biological recurrence (Table 1). The median dose received by 50%/1% of the rectum and bladder by this prior treatment were 44.1 (range, 60.0 - 38.5)/71.0 (range, 74.5 - 62.4) and 59.0 (range, 67.2 - 43.4)/74.0 (range, 78.0 - 64.4) Gy, respectively. The median rectal volume receiving 35 Gy was 79.4%, and range from 56.0 to 96.0%. Local relapse was proven by PET-CT examination with ^18^F-choline; failures were confirmed by sextant biopsy in all but one patient. A positive correlation between ^18^F-choline uptake and the location of the histological proven recurrence was observed in all 6 patients. Table 2 details the radiological and pathological correlation of these recurrences. PET/CT imaging was performed on the Biograph 16 scanner (Siemens Medical Solution, Erlangen, Germany) operating in 3D mode (Fig. 1). An endorectal MRI, with spectroscopy and contrast enhancement, was acquired for all patients [10]. The main organs at risk (OARs) considered for all patients were the urethra (defined on the base of MR imaging and verified by an experienced radiologist), bladder, rectum, penile bulb and femoral heads The non-target tissue was defined as the patient's volume covered by the CT scan minus the planning target volume (PTV).

**Table 1:** Patients characteristics

**No of patient**	**1**	**2**	**3**	**4**	**5**	**6**	**7**
Age (years)	81	63	79	69	77	78	69

Recurrence time (years)	5.86	4.82	6.75	5.16	5.85	5.82	7.55

PSADT (month)	13.9	9.0	10.2	8.5	10.2	5.5	25.8

Tumour stage (at relapse)	T2c	T3b	T2c	T2c	T3a	T2c	T3b

PSA at recurrence (ng/ml)	5.11	6.76	2.80	5.14	6.32	5.95	13.00

Gleason score at recurrence	3+4	-	3+4	3+3	4+3	4+3	3+3

GTV (cm^3^)	0.61	1.09	3.48	5.08	5.75	10.36	19.93

CTV (cm^3^)	2.59	3.29	9.72	12.84	15.65	20.91	38.61

PTV(cm^3^)	6.68	8.13	22.13	26.67	30.42	39.47	64.20

Abbreviations: PSA = prostate-specific antigen; PSADT = prostate-specific antigen doubling time; PET-CT = Positron Emission Tomography and Computed Tomography; GTV = gross tumour volume; CTV = Clinical Target Volume; PTV = Planning Target Volume.

**Table 2:** Prostate cancer recurrence on MRI, PET and biopsy

	**Recurrent Site**		
	
**No of patient**	**MRI**	**PET CT**	**Biopsy (Number of positive cores)**
1	L, R	L	L (1/7); R (0/6)

2	SV	SV	ND

3	L, R	L, R	L (1/3); R (3/4)

4	R	R	L (0/4); R (3/4)

5	L	L, R	L (2/3); R (1/3)

6	L, R	L, R	L (3/3); R (4/4)

7	L, R, SV	L, R, SV	L (4/4); R (3/4)

Abbreviations: L, left prostate lobe; R, right prostate lobe; SV, seminal vesicle; ND, not done.

For all patients, GTV was outlined using the signal-to-background ratio-based adaptive thresholding technique described in [11] and adapted to our PET/CT scanner characteristics. Data acquisition and processing protocols are described elsewhere [12]. The clinical applicability of detecting prostate recurrence with ^18^F-Choline PET has been demonstrated in our previous series [13]. Fig. 1 depicts the PET GTV for 1 patient. Clinical target volume (CTV) was defined adding a 3D anisotropic margin of 3 mm (CTV was however limited to the prostate and seminal vesicles and could not be stretched beyond these structures), excluding the urethra in all cases. PTV was defined adding a 3D anisotropic margin of 3 mm (2 mm in proximity of the urethra) to the CTV. A summary of the sizes of the GTVs, PTVs and OARs are detailed in Tables 1 and 2.

Dose prescription of 56 Gy to PTV was delivered according to a hypofractionated radiation schedule consisting of 14 daily fractions of 4 Gy, twice weekly (overall treatment time, 7 weeks) [14]. All plans were normalized to the mean dose of the PTV.

Plans aimed to cover at least 95% of the PTV with a dose greater than 90% of the dose prescription. An over-dosage of maximum 61.6 Gy (110%) was allowed to 5% of both CTV and PTV. For the urethra, the maximum dose was constrained to 37 Gy. A dose lower than 28 Gy delivered to 50% of the volume of the bladder, penile bulb and femoral heads was required for these OARs; likewise, a dose < 17 Gy was constraint to 30% of the rectal volume.

Four sets of plans were compared in this study, all designed on the Varian Eclipse treatment planning system (version 8.6.10) with 6 MV photon beams from a Varian Clinac equipped with either a Millennium Multileaf Collimator (MLC) with 120 leaves (RA_M120; spatial resolution of 5 mm at isocentre) or a High Definition MLC with 120 leaves (RA_HD120; spatial resolution of 2.5 mm at isocentre). Plans for RA were optimized selecting a maximum DR of 600 MU/min and a fixed DR of 600 MU/min was selected for IMRT.

The Anisotropic Analytical Algorithm photon dose calculation algorithm was used for all photon cases [15]. The dose calculation grid was set to 2.5 mm.

### RA

RA uses continuous variation of the instantaneous dose rate, MLC leaf positions and gantry rotational speed to optimize the dose distribution. Details about RA optimization process have been published elsewhere [8]. To minimize the contribution of tongue and groove effect during the arc rotation and to benefit from leaves trajectories non-coplanar with respect to patient's axis, the collimator rotation in RA remains fixed to a value different from zero. In the present study collimator was rotated to ~30° depending on the patient.

For the study, two sets of plans were optimized, each with a single arc 360°. The first set (RA_M120) was created using the Millennium MLC, the second set (RA_HD120) with the High Definition MLC.

### IMRT

Plans were designed according to the dynamic sliding window method [16] with five fixed gantry beams. One single isocentre was located at the target center of mass. All beams were coplanar with collimator angle set to 0°. The Millennium MLC was used for the study.

### IMPT

Intensity modulated proton plans were obtained for a generic proton beam through a spot scanning optimization technique implemented in the Eclipse treatment planning system from Varian. The optimization process has been detailed elsewhere [17]. Spot spacing was set to 3 mm, circular lateral target margins were set to 5 mm, proximal margin to 5 mm and distal margin to 2 mm. In all cases coplanar beam arrangement was adopted using 3 fields, one with posterior and two with anterior oblique incidence.

Quantitative evaluation of plans was performed by means of standard Dose-Volume Histogram (DVH). For GTV and PTV, the values of D_98% _and D_2% _(dose received by the 98% and 2% of the volume) were defined as metrics for minimum and maximum doses and thereafter reported. To complement the appraisal of minimum and maximum dose, V_95% _and V_107% _(the volume receiving at least 95% or at most 107% of the prescribed dose) were reported. The homogeneity of the treatment was expressed in terms of the standard deviation and of D_5%_-D_95%_. The conformality of the plans was measured with a Conformity Index, CI_90% _defined as the ratio between the patient volume receiving at least 90% of the prescribed dose and the volume of the PTV.

For OARs, the analysis included the mean dose, the maximum dose expressed as D_1% _and a set of appropriate volume (V_X_) and dose (D_Y_) metrics.

For non-target tissue, the integral dose, (Dose_Int_) is defined as the integral of the absorbed dose extended to over all voxels excluding those within the target volume (Dose_Int _dimensions are Gy*cm^3^). This was reported together with the observed mean dose and some representative V_x _values.

To visualize the global difference between techniques, average cumulative DVH for GTV and PTV, OARs and healthy tissue, were built from the individual DVHs. These DVHs were obtained by averaging the corresponding volumes over the whole patient's cohort for each dose bin of 0.05 Gy.

To appraise the difference between the techniques, the paired, two-tails Student's *t*-test was applied whenever applicable. Data were considered statistically significant for p < 0.05.

## Results

The mean prostate volume was 35.4 ± 7.8 cm^3 ^and the average GTV and PTV volumes are reported in Table 3. The mean ratio between PTV and prostate volume was 0.77 ± 0.50 with a range from 0.19 to 1.76.

**Table 3:** Dosimetric results for GTV and PTV

**Parameter**	**IMRT**	**IMPT**	**RA_HD120**	**RA_M120**	***p ***
**GTV Volume [cm3] 6.7 ± 6.8 [0.6-19.9]**					

Mean [Gy]	58.9 ± 2.2	56.5 ± 1.0	57.2 ± 0.6	57.3 ± 0.8	e

D_5_-D_95 _[Gy]	12.4 ± 6.9	12.5 ± 6.0	8.5 ± 5.3	10.2 ± 5.3	a,b,c,d,e,f

D_2 _[Gy]	64.6 ± 1.2	61.9 ± 2.7	60.7 ± 2.0	61.5 ± 1.6	a,b,c,d

D_98 _[Gy]	49.3 ± 7.7	46.6 ± 6.9	49.2 ± 6.6	48.2 ± 6.3	d,e,f

V_95 _[%]	88.6 ± 10.8	88.9 ± 10.5	92.6 ± 7.9	91.4 ± 8.5	d,e,f

V_107 _[%]	52.3 ± 27.8	21.1 ± 14.9	9.1 ± 12.1	19.3 ± 14.2	b,f

**PTV Volume [cm3] 27.7 ± 19.6 [6.7-64.2]**					

Mean [Gy]	56.0 ± 0.0	56.0 ± 0.0	56.0 ± 0.0	56.0 ± 0.0	

D_5_-D_95 _[Gy]	15.0 ± 2.0	13.6 ± 4.3	11.8 ± 2.7	13.2 ± 3.2	a,b,c,d,f

D_2 _[Gy]	63.6 ± 0.9	61.4 ± 1.6	60.7 ± 1.5	61.5 ± 1.3	a,b,c,d,f

D_5 _[Gy]	62.3 ± 0.9	60.7 ± 1.4	60.0 ± 1.2	60.7 ± 1.2	a,b,c,d,f

D_98 _[Gy]	43.8 ± 2.8	42.4 ± 5.4	44.1 ± 4.0	43.5 ± 4.5	d,e,f

V_95 _[%]	77.2 ± 2.2	85.6 ± 5.0	83.7 ± 3.3	81.8 ± 4.2	a,b,e,f

V_107 _[%]	18.2 ± 2.6	12.6 ± 8.5	6.9 ± 6.4	12.5 ± 8.6	b,d,f

a = IMRT vs IMPT b = IMRT vs RA_HD120 c = IMRT vs RA_M120

d = IMPT vs RA_HD120 e = IMPT vs RA_M120 f = RA_HD120 vs RA_M120

For the GTV and PTV, the RA_HD120 and IMRT techniques produced the best and worst dose homogeneity, respectively (Table 3). The GTV coverage was optimal with RA (mean V_95% _92%; Table 3). The PTV coverage (V_95%_) was better with IMPT, intermediate with RA and worse with IMRT (Table 3).

The GTV and PTV V_95%_-difference observed between RA_HD120 and RA_M120 (Table 3) is due to different MLC characteristics, namely spatial resolution and transmission. IMPT showed a moderate improvement compared to IMRT (V_107 _and V_95_; Table 3). Interestingly, IMPT did not reach the performance of RA_HD120 for V_107 _for both the GTV and PTV (Table 3). None of the techniques achieved the planning objective on minimum PTV dose (Table 3). IMRT failed to reach the objective on D_5% _for PTV while all others met the condition (Table 3).

**Figure 2:**
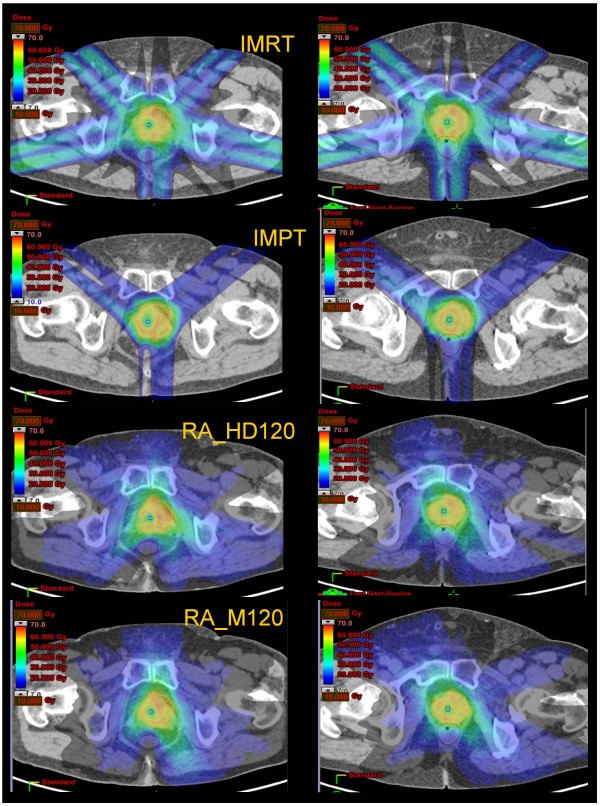
**Color wash IMRT, IMPT, RA_HD120 and RA_M120 dose distributions for the planning target volume (PTV) for two patients with recurrent prostate cancer**.

**Figure 3:**
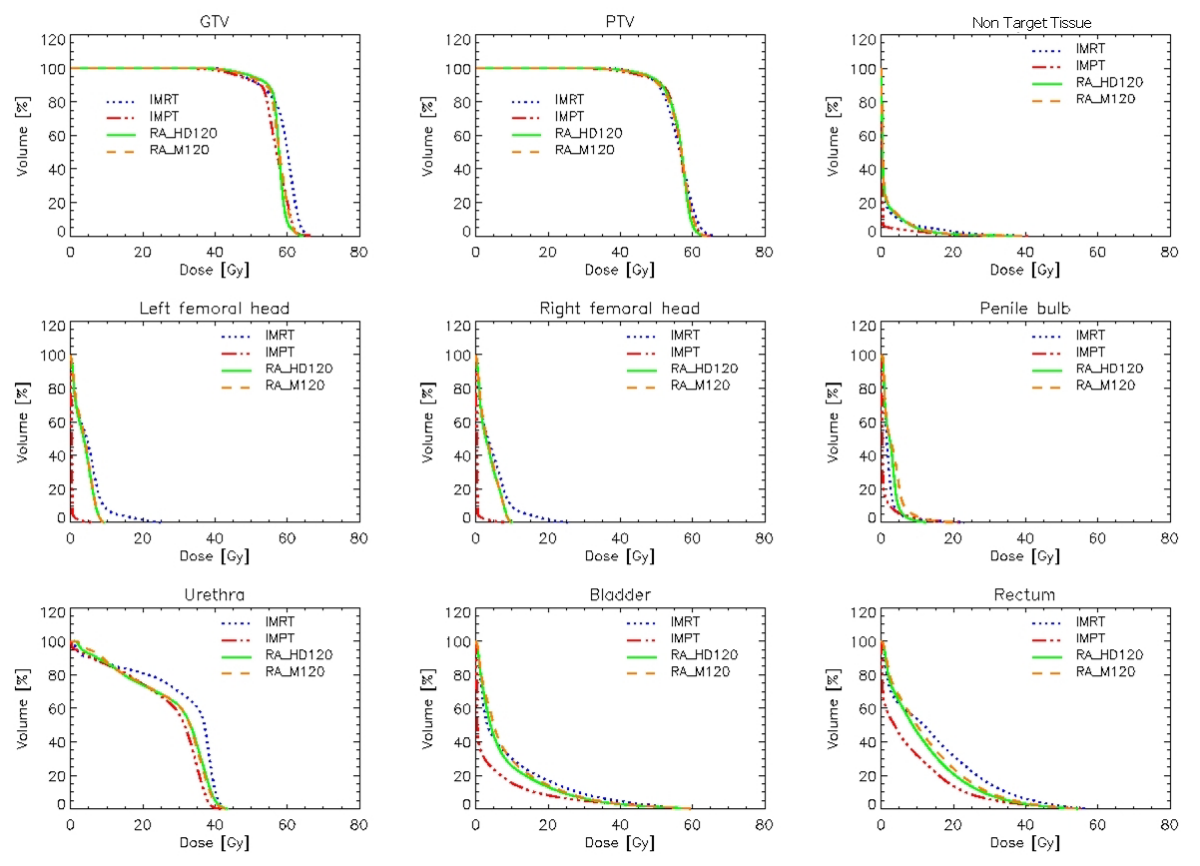
**Mean DVHs for CTV, PTV and OARs**.

The rectal dose was significantly decreased with IMPT and RA, respectively (Fig. 2, 3). For the intermediate dose level, these two techniques more than halved the percentage of rectal volume receiving 35 and 45 Gy (Table 4). For the high-dose level, IMPT delivered a decreased dose when compared to the other two photons techniques (Table 4).

**Table 4:** Dosimetric results for OARs and non target tissues

**Parameter**	**IMRT**	**IMPT**	**RA_HD120**	**RA_M120**	***p ***
**Rectum. Volume [cm3] 48.6 ± 17.6 [28.4-72.5]**					

D_50 _[Gy]	10.1 ± 6.2	4.1 ± 4.0	8.2 ± 3.9	9.1 ± 4.2	a,b,d,e,f

D_1 _[Gy]	49.6 ± 6.8	45.1 ± 9.2	45.2 ± 8.3	46.5 ± 7.8	a,b,c

V_35 Gy _[%]	9.8 ± 5.3	3.9 ± 2.7	5.1 ± 3.0	5.9 ± 3.3	a,b,c,e

V_45 Gy _[%]	3.6 ± 2.4	1.6 ± 1.3	1.6 ± 1.1	1.9 ± 1.3	a,b,c

**Urethra. Volume [cm3] 0.7 ± 0.1 [0.6-0.8]**					

D_50 _[Gy]	31.4 ± 13.1	26.8 ± 11.7	28.6 ± 11.4	28.6 ± 10.9	a,b,c,d,e

D_1 _[Gy]	40.1 ± 3.3	38.1 ± 2.4	39.8 ± 3.5	39.3 ± 3.3	a,c,d,f

V_35 Gy _[%]	60.7 ± 41.7	25.1 ± 21.1	38.0 ± 25.3	36.0 ± 24.0	a,b,c

V_40 Gy _[%]	11.0 ± 12.8	0.6 ± 1.1	5.1 ± 5.4	4.0 ± 5.6	

**Left femoral head Volume [cm3] 60.1 ± 4.4 [54.8-67.6]**					

D_50 _[Gy]	3.9 ± 2.6	0.1 ± 0.1	3.3 ± 2.1	3.5 ± 2.1	a,b,d,e,f

D_1_Gy]	14.6 ± 7.2	2.3 ± 2.0	7.4 ± 1.5	7.6 ± 1.3	a,b,c,d,e

**Right femoral head Volume [cm3] 60.9 ± 5.8 [54.6-71.6]**					

D_50 _[Gy]	3.9 ± 2.7	0.1 ± 0.1	3.2 ± 2.3	3.4 ± 2.1	a,d,e

D_1_Gy]	15.3 ± 7.5	2.5 ± 3.0	8.0 ± 1.8	8.0 ± 1.7	a,b,c,d,e

**Bladder. Volume [cm3] 109.8 ± 63.6 [32.7-234.2]**					

D_50 _[Gy]	4.9 ± 3.2	0.7 ± 0.9	4.6 ± 2.6	5.2 ± 3.0	a,d,e,f

D_1 _[Gy]	42.3 ± 17.0	38.8 ± 19.6	41.3 ± 16.3	42.1 ± 15.8	

V_35 Gy _[%]	6.4 ± 6.3	3.9 ± 4.3	4.1 ± 4.1	4.5 ± 4.2	a

V_50 Gy _[%]	1.9 ± 2.7	1.4 ± 2.1	1.3 ± 2.1	1.3 ± 2.1	

**Penile bulb. Volume [cm3] 7.2 ± 3.2 [3.0-13.2]**					

D_50 _[Gy]	2.0 ± 1.5	0.9 ± 1.4	2.5 ± 1.7	3.2 ± 2.5	a,b,c,d,e

D_1 _[Gy]	7.6 ± 9.4	7.1 ± 9.0	5.8 ± 4.6	7.7 ± 7.4	

**Non Target Tissue**					

Mean [Gy]	2.0 ± 0.8	0.7 ± 0.3	1.8 ± 0.7	1.9 ± 0.7	a,b,d,e,f

V_10 Gy _[%]	6.0 ± 2.6	2.8 ± 1.3	4.7 ± 2.5	5.1 ± 2.8	a,b,c,d,e

CI_90_	1.3 ± 0.1	1.6 ± 0.2	1.3 ± 0.1	1.3 ± 0.1	a,d,e

Dose_Int _[Gy*cm^3 ^10^4^]	3.3 ± 1.6	1.1 ± 0.5	2.9 ± 1.3	3.1 ± 1.4	a,b,d,e,f

a = IMRT vs IMPT b = IMRT vs RA_HD120 c = IMRT vs RA_M120

d = IMPT vs RA_HD120 e = IMPT vs RA_M120 f = RA_HD120 vs RA_M120

For the urethra, none of the techniques was able to keep the maximum dose below the threshold of 37 Gy (Table 4). IMPT violated this dose level by approximately 1 Gy, while RA and IMRT exceeded this metric by 2.3 - 2.8 and 3 Gy, respectively. For the intermediate dose level, IMPT and RA approximately halved the percentage of urethral volume receiving 35 and 45 Gy (Table 4), respectively. Since the urethra was included in the PTV in a majority (5/7) of patients, the observed values were expected.

IMPT resulted in an almost complete avoidance of femoral heads (Fig. 2; median inferior to 0.1 Gy; Table 4) while both RA reduced maximum dose of about 50% compared to IMRT.

IMPT was the best technique to spare the penile bulb (Fig. 3). For the bladder, all non-IMPT techniques were identical (Table 4; Fig 3).

Non target tissue irradiation was limited for all techniques and the mean dose was kept under the Gy unit for the majority of patients (Table 4). IMPT showed a Dose _Int _of approximately a factor 3 lower than all the photon techniques. The CI was however better with photons techniques (mean CI improvement: 18%), because of the wider lateral and distal spread induced by spot size, spacing and margins used to achieve sufficient target coverage (Table 4).

For all but one OARs (urethra), RA_HD120 results were better than those observed with RA_M120 (Table 4). This observed OAR's sparing derives from the superior spatial resolution and inferior transmission through leaves with the former when compared to the latter technique. RA_M120 generally improved OARs sparing compared to IMRT suggesting, given the usage of same MLC, a superior modulation capability (Table 4). The only exception in this pattern is represented by the penile bulb (D_1 _7.7 *vs*. 7.6; Table 4). This OAR is moderately distant from the target and affected by higher scattering, mostly compensated if the High Definition HD_120 MLC is used instead of the Millennium M120.

## Discussion

More than one out of four patients presenting a BF after definitive RT will have clinical evidence of local recurrence within 5 years [18]. Failure to control the prostate is not only a cause of local disease progression but provides possibly a nidus for systemic spread, as shown by the distant metastasis rate in this population [18]. A body of literature predicts however that complications, not limited to but including, the rectum [19,20] and urethra [21,22], after any salvage local therapy in a post-RT setting, is significant. As such, rectal and urethral toxicity is a major concern when using external beam RT as salvage local therapy [23]. We have undertaken a treatment plan comparative study to assess the dose deposition to these OARs, using intensity modulated photons and protons techniques. Overall, IMPT and RA techniques substantially decreased the dose in the intermediate range level to the rectum and urethra (Fig. 3). All the volume and dose metrics for these OARs were substantially decreased with IMPT and RA when compared to IMRT (Table 4). As such, these findings might have bearing on clinical practice for recurrent prostate cancer after RT. RA or IMPT might be an alternative to salvage prostatectomy, cryosurgery or brachytherapy in a selected number of patients.

Non conventional RT, be it IMRT, IMPT or RA, was simulated essentially to capitalize the prerequisite tight dose conformation necessary to administer radiation to these heavily pre-treated prostates. This conformal ability was coupled with the theoretical advantage of hypo fractionation in prostate cancer, while respecting the dose-tolerance of pre-irradiated OARs in the vicinity of the prostate. An increasing body of data now suggests that the α/β ratio for prostate is low, possibly in the range of 1-3 Gy [24]. If this metric is accurately low, then hypo fractionated radiation schedules should improve the therapeutic ratio [25]. It was chosen to elect a hypo fractionated radiation schedule for this treatment plan comparison as the dose limiting OARs in vicinity of the GTV was a major issue and may have α/β ratios exceeding that for prostate cancer, thus decreasing the probability of toxicity and increasing the probability of cure. Assuming a complete inter-fraction complete repair and no time factor, the total equivalent dose of 56 Gy delivered in 14 fractions would be about 88 Gy if the α/β ration is 1.5 if delivered at 1.8 Gy/fraction, according to the presumed α/β ratio for prostate cancer using the linear quadratic model.

Biochemical control of prostate cancer patients with recurrent disease may ultimately not be achieved for two main reasons. First, the biochemical failure might be related to the presence of occult metastasis at salvage treatment. It is therefore of paramount importance to appropriately choose patients who are most likely to have local disease only, not limited to but including, interval PSA failure > 3 years, positive re-biopsy, low Gleason score at re-biopsy, low PSA values at relapse, PET positive intra-prostatic tumor, negative bone scan/pelvic imaging studies and PSA-DT > 8 months. All our patients presented these characteristics for the 6 former factors (1 re-biopsy medically contra-indicated) and all but 1 had a PSA-DT > 8 months [26,27] (Table 1). Second, the local disease may be inadequately addressed by conventional radiology. Unfortunately, approximately half of all patients will have extraprostatic disease [28] and it is thus critical to optimally define the target volume. It is axiomatic that any suboptimal GTV and PTV delineation may ultimately translate into local failure. For all patients, we have used metabolic imaging in conjunction with endo-rectal MRI. PET imaging with the non-FDG tracers, such as ^11^C-choline, ^11^C-acetate, and ^18^F-fluorocholine have shown promising results [29]. Notwithstanding the spatial limitation of PET for the staging of prostate cancer (i.e. capsule invasion, cT_3_), ^18^F-choline PET has shown an overall sensitivity of 86% in detecting local recurrent disease in a recent series [30]. Likewise, Reske *et al*. [31] assessed the value of choline PET/CT for localizing occult relapse of prostate cancer after radical prostatectomy in 49 patients. Focally increased ^11^C-choline uptake in the prostatic fossa was observed in 70% of patients with histological verification of recurrence. As such, any re-irradiation techniques should deliver radiation to small morphologically and metabolically defined GTV.

Patient selection for re-irradiation according to clinical and biochemical factors is of critical importance as discussed earlier. First, the physicians have to comprehensively assess the type of failure of her/his recurrent prostate cancer. Second, the site of local failure has to be defined precisely using biopsy and PET CT. Of note, in our small cohort, all patients had a morphological-metabolic and -pathological correlation (Table 2). None less central to treatment success are the tumor geometrical characteristics and localization within the prostate. All our patients presented with small local recurrences, with a mean GTV and PTV of 6.6 and 28.2 cm^3^, respectively (Table 1). The smaller the tumor, the easier it will be to meet appropriately the OAR's dose constraints for re-irradiation. The 3-D locations of these recurrent tumors were however challenging. The urethra was in all but two cases fully surrounded by the GTV. Huang *et al*. have reported on 47 salvage prostatectomies performed in prostate cancer patients treated with primary RT. Sixty-seven % of patients had recurrent cancer ≤ 5 mm from the urethra [28]. This OAR, and not the rectum, was the dose limiting structure in a recent HDR brachytherapy series [23]. This necessitates the application of the most advanced radiation techniques to guarantee satisfactory OAR's conformal avoidance.

All techniques were able to deliver high-dose hypo-fractionated re-irradiation. Cumulatively, IMRT, compared to IMPT or RA, appeared to be less optimal, when certain but not all dosimetric parameters are analyzed (Table 3, 4). The magnitude of the clinical benefit of these latter techniques remains however to be demonstrated. The less favorable IMRT plan comparison metrics results of inferior OAR sparing and of higher target dose heterogeneity and significantly higher GTV and PTV hot spots (Fig. 3).

As expected, IMPT, presented a significantly better sparing of non target tissues but did not offered a substantial improvement of target coverage compared to RA. The usage of the High Definition MLC for RA is somehow advantageous compared to the Millennium MLC for both target and OARs. This fact is noticeable and logical, given the very small size of the GTVs and PTVs. This observed difference between RA_HD120 and RA_M120 may also be clinically not pertinent. RA, with the most generally available Millennium MLC might therefore be considered appropriate also for very small GTVs, offering this modality to a wider number of patients.

Another objective was to assess the capability of the different radiation techniques to manage demanding and opposite planning objectives such as PTV coverage *vs*. urethra sparing. Such a dosimetric challenge, given the relative position of the two volumes, requires the generation of very steep dose gradients to create in an ideally uniform dose distribution of 56 Gy a donut hole with a maximum dose of about 67% (a step of about 20 Gy in 2-3 mm, i.e. 6-10 Gy/mm). Although all techniques have failed these paradoxical dose-constraints, IMPT and RA techniques could be considered appropriate for these challenging patients (Table 4; Fig. 2). These data are supportive of the sophisticated modulation capabilities of RA with one single arc, despite recent criticisms raised on the basis of over-simplified geometrical assumptions [32].

There were several limitations of our study. First, the small sample size limits the applicability of our conclusions to all prostate cancer patients with recurrent local disease after RT. As only 25% of these patients could be eligible to local curative treatment [33], clinical judgment (i.e. patient's overall health, morbidity from the local treatment, recurrent tumor characteristics) should always supersede any institutional re-treatment protocols applied indiscriminately to this population. Second, it is axiomatic that any high-dose re-irradiation of the prostate should be undertaken only with appropriate treatment positioning protocols, not limited but including image guidance radiation delivery, robotic couch positioning and prostatic implants for optimal radiation targeting. These issues were purposely not addressed in this dose-comparative study. Third, the localization of the urethra on the planning CT can be problematic, even with the help of an experienced radiologist and CT-MRI fusion. It may be appropriate to catheterize these challenging patients with small catheters during RT simulation. Fourth, only generically dose constraints for OARs were implemented for the RT planning of recurrent prostate cancer in this series. At this juncture, given the potential re-irradiation-induced toxicity, consideration could be given to the prior individual RT plan to adapt each re-treatment plans. As such, given the dosimetric metrics of the prior RT, some patients could possibly not be retreated with these techniques. Finally, the issue of delivering radiation with a high dose gradient (i.e. 6 - 10 Gy/mm) to PET defined GTVs has not been addressed in this study. This concern will be developed in a future publication.

## Conclusion

RA, IMPT and IMRT techniques were compared for salvage local treatment in patients with recurrent prostate cancer after RT. All techniques proved to be dosimetrically adequate, with IMPT offering the best sparing of OARs and RA a slightly superior coverage of GTV with an OAR sparing intermediate between IMRT and IMPT. Given limited accessibility of proton facility, RA appears to be a promising treatment solution for particularly small recurrent prostate tumors.

## Abbreviations

RA: volumetric modulated arcs radiation therapy; IMRT: intensity modulated radiation therapy; RT: radiation therapy; IMPT: intensity modulated proton therapy; GTV: recurrent gross tumor volume; PET: positron emission tomography; BF: biochemical failure; DVH: dose volume histogram; CI: conformity index.

## Competing interests

LC acts as Scientific Advisor to Varian Medical Systems and is Head of Research and Technological Development to Oncology Institute of Southern Switzerland, IOSI, Bellinzona. Other authors have no conflict of interest.

## Authors' contributions

RM, LC and DCW were responsible for the primary concept and the design of the study; HW, HV, HZ and LC performed the data capture and analysis; LC performed the statistical analysis; DCW and LC drafted the manuscript; DCW and HW reviewed patient data; all authors revised and approved the final manuscript.
